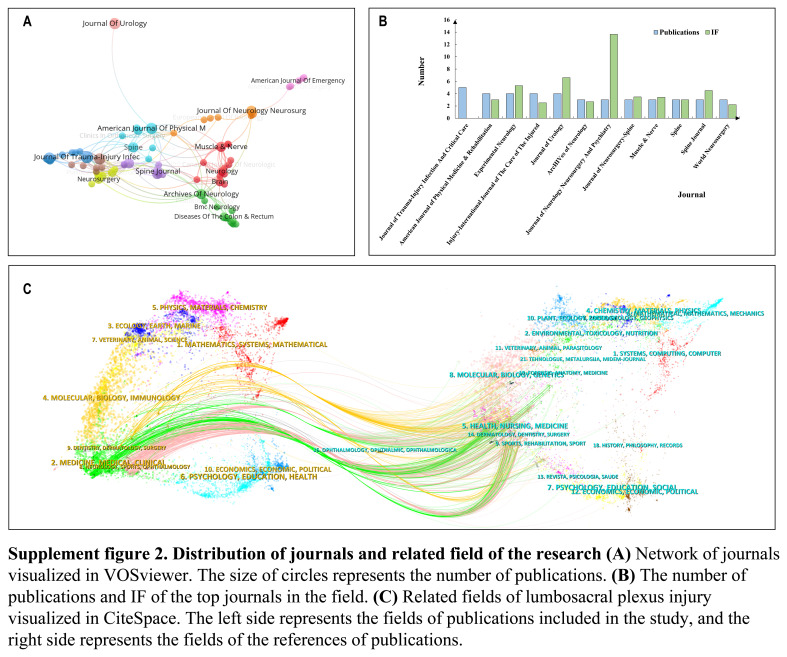# Could nerve transplantation be the future of this field: a bibliometric analysis about lumbosacral plexus injury

**DOI:** 10.1097/JS9.0000000000001332

**Published:** 2024-03-21

**Authors:** Sheng Wang, Demeng Xia, Danyan Song, Nan Lu, Aimin Chen

**Affiliations:** a Department of Traumatic Orthopedics, Shanghai Fourth People’s Hospital, School of Medicine, Tongji University; b Department of Pharmacy, Seventh People’s Hospital of Shanghai University of Traditional Chinese Medicine; c Emergency Department, Changhai Hospital, Naval Medical University, Shanghai, People’s Republic of China

**Keywords:** lumbosacral plexus injury, nerve transfer, repair mechanism, sacral neuromodulation

## Abstract

**Background:**

Lumbosacral plexus injury is a highly distressing clinical issue with profound implications for patients’ quality of life. Since the publication of the first relevant study in 1953, there has been very limited progress in basic research and clinical treatment in this field, and the developmental trajectory and research priorities in this field have not been systematically summarized using scientific methods, leaving the future direction of this research to be explored.

**Methods:**

Utilizing publications from the Web of Science (WoS) database, our research employed bibliometric methodology to analyze the fundamental components of publications, synthesize research trends, and forecast future directions.

**Results:**

A total of 150 publications were included in our study, and the impressive advancement of research heat in this field can be attributed to the continuous increase in the number of papers, ranging from 14 papers in 2000 to 34 papers in 2023 over 5 years. Regarding the country, a central position in both quantity (H-index=125) and quality of publications (65 publications) is occupied by the United States, and close collaborations with other countries are observed. In terms of publication institutions, the highest number of publications (nine publications) is held by the Second Military Medical University. The journal with the most publications (five publications) is the Journal of Trauma-Injury Infection and Critical Care. A pivotal role has been played by basic medical research in the development of this field. Concerning hotspots, the focus of the research core can be divided into three clusters (etiology, diagnosis and treatment; molecular, cells and mechanisms; physiology, and pathology).

**Conclusion:**

This marks the inaugural bibliometric analysis of lumbosacral plexus injuries, offering a comprehensive overview of current publications. Our findings illuminate future research directions, international collaborations, and interdisciplinary relationships. Future research will emphasize clinical treatment and mechanism research, with a focus on sacral nerve stimulation and nerve transplantation.

## Introduction

Highlights

- The research attention in lumbosacral plexus injury will continue to grow in the future.
- The cooperation between countries and disciplines is important in the development of this field.
- Future research direction is focused on clinical and treatment and mechanism.
- Key points of future research in this field are nerve transfer and sacral neuromodulation.

The lumbosacral plexus plays a critical role in controlling lower limb movement, sensory functions, excretion, and sexual function. Comprising the lumbosacral plexus root (L1-L5) and sacral plexus root (L4-S4), this intricate structure is discreetly located in front of the sacroiliac joint before entering the pelvic cavity. Its deep anatomical position and complex structure make it susceptible to injury, often resulting in complete loss of lower limb motor function, accompanied by sensory and urinary and bowel dysfunction^1^. While trauma-induced lumbosacral plexus injuries are relatively uncommon due to the protective shield of muscles and bones, this protection also poses significant treatment challenges^2^. Furthermore, in recent years, lumbosacral plexus injuries have been on the rise due to various factors such as lower limb nerve blocks, surgical mishaps, pelvic radiation therapy, and poisonings^3,4^. These diverse causes can cause severe damage to the lumbosacral nerves, leading to a lifetime of lower limb motor and sensory pain, as well as urinary and bowel disorders, substantially diminishing the quality of life for affected individuals. Consequently, research in this field holds crucial clinical and practical significance.

Bibliometrics, as a scientific method for analyzing subjects within a field, offers an invaluable tool. Utilizing software like VOSviewer and CiteSpace, bibliometrics processes publication data systematically, enabling precise visualization of research content. This approach aids in swiftly grasping research trends and hotspots within a given field^5^. Furthermore, it allows for reasonable predictions of research outcomes, guiding scholars toward specific research directions^6^. By integrating and processing bibliometric information, comprehensive discussions within a field become more scientific and rigorous. Consequently, bibliometrics has gained increasing attention and application in recent years.

However, it is worth noting that conclusive studies on lumbosacral plexus injuries are currently lacking, and most of the existing literature in databases consists of case reports. To address this gap, we have chosen to employ the bibliometrics method to study this field, with the aim of providing a comprehensive overview and guiding future research directions for clinicians involved in the treatment of lumbosacral plexus injuries.

## Materials and methods

### Data screening and inclusion

**Figure 1:**
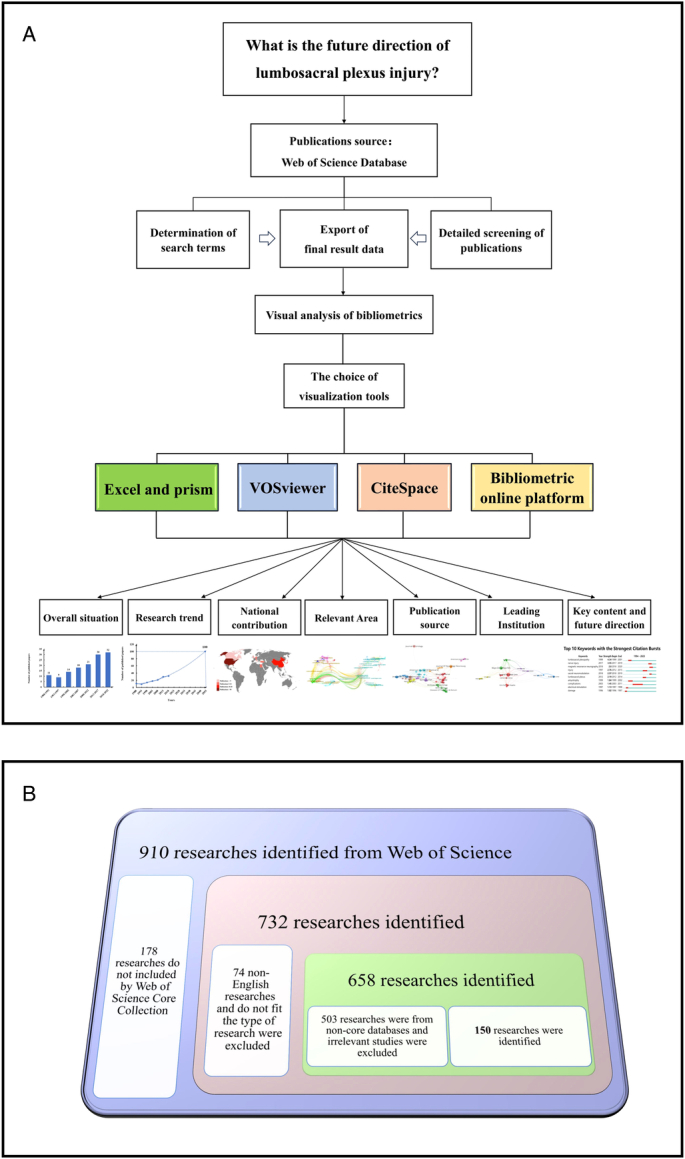
Flow diagram of the research process (A) research process. (B) The specific process of screening publications.

Ensuring a sufficient sample size from a reliable database is crucial for the accuracy of research in any field. The WoS database is a highly reputable source that includes medical journals^7^. Therefore, we selected the WoS Core Database as our primary data source. The detailed process is depicted in Figure 1A. Initially, we formulated the search query as follows: TS=Lumbosacral nerve injury (Topic) or Lumbosacral plexus injury (Topic) or Lumbosacral nerve injuries (Topic) or Lumbosacral plexus injuries (Topic). This search yielded a total of 910 publications, with 732 of them present in the WoS Core Database. Subsequently, we excluded non-English publications, duplicates, and publications that did not meet the specified criteria. A total of 658 publications met the inclusion criteria. Furthermore, we conducted a manual review to ensure that the selected publications were directly relevant to lumbosacral nerve injuries. This review process involved two authors (WS and XDM), with any discrepancies being resolved by the experienced corresponding author (CAM). Ultimately, 150 publications were retained for our analysis. The screening process is outlined in Figure 1B.

### Bibliometrics analysis

Bibliometrics is a methodology for analyzing research directions and potential hotspots within a specific research field, relying on effective software tools for analysis. Notably, Prism and Excel are employed for creating fundamental statistical charts, while VOSviewer facilitates in-depth analysis of national contributions, institutional contributions, and publication keywords^8^, and CiteSpace, on the other hand, is primarily used to categorize related fields and identify burst words^9^. Both tools excel in presenting information visually. To enhance the clarity of our results, we utilized an online bibliometric analysis platform to assess international relationships. In this study, the key aspects encompass the overall publication trends, country contributions and collaboration networks, contributions from publishing institutions, publication sources, research field categorization, keyword clustering, and identification of future research hotspots.

### Mining and summary of future research hotspots

Building upon the bibliometric analysis of fields and research trends, we conducted a comprehensive review of pertinent publications. This allowed us to delve deeply into the focal points and emerging areas of research on lumbosacral plexus injury. We summarized existing research findings to provide a foundation for future studies in this field.

## Results

### Overall publication trend (research popularity)

**Figure 2:**
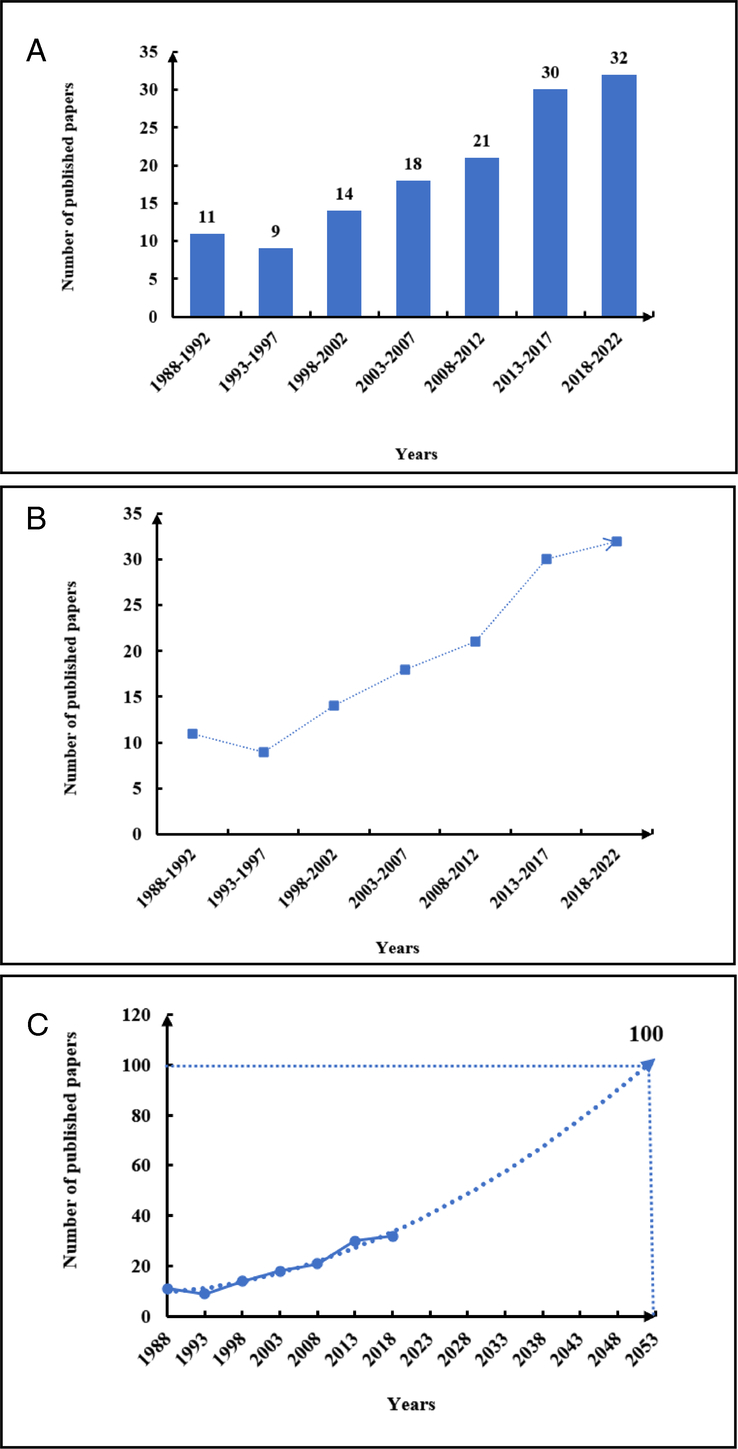
Flow diagram of overall publication trend (A) The number of publications in this field in the last 35 years. (B) Changes in the trend of publication in the last 35 years. (C) Research trends in this field in the next 30 years.

The number of published papers serves as the most indicative measure of research popularity. Our analysis of publication data reveals that the largest number of papers were published in the 5-year period from 2018 to 2022, with a total of 32 papers. This figure is double the number of papers published in the 5 years from 1988 to 1992 (Fig. 2A). Overall, there is a consistent upward trend in the number of published documents each year (Fig. 2B). Projecting this development trend forward, we anticipate that by 2053–2057, the total number of papers published over 5 years will surpass 100 (Fig. 2C). This trend underscores the gradual increase in research popularity within this field over the next 30 years.

### National contributions and partnerships

**Figure 3:**
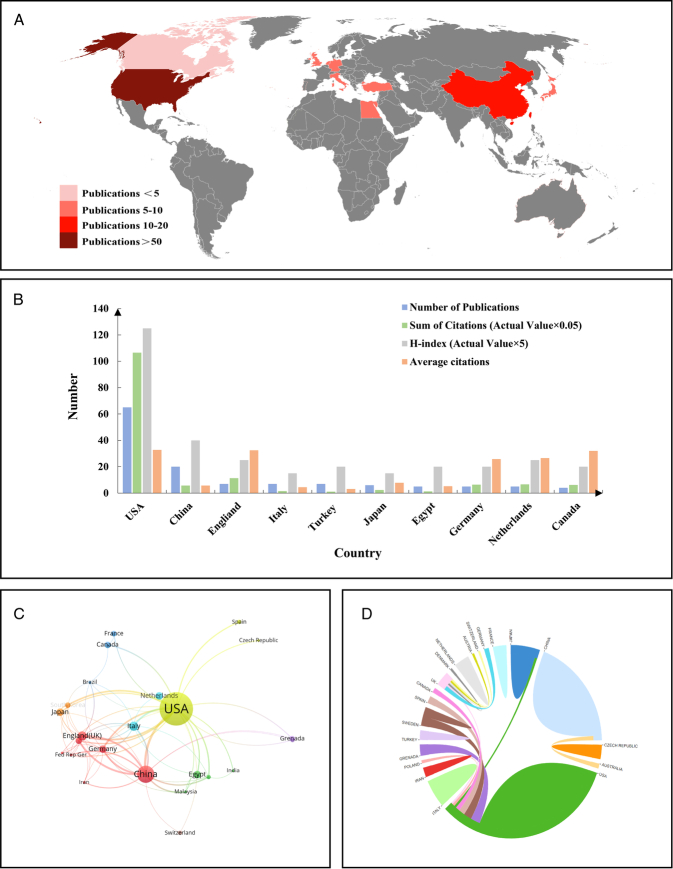
Contributions of different countries to the research. (A) Overall situation of number of publications worldwide. (B) Number of publications, citation frequency (×0.05), H-index (×5), and average citations in the top 10 countries or regions. (C and D) The number of national publications and the cooperative relations between them.

Our visual analysis, based on the number of publications by each country, highlights the United States as the leading contributor in terms of the sheer number of articles published. While other countries also make contributions, their output is comparatively lower (Fig. 3A). As the top publishing nation with 65 articles, the United States holds the first position in both the H-index of all articles (125) and the average number of citations per article (32.77). Interestingly, the United Kingdom secures the second position in the average number of citations per article (32.43), even though it ranks lower in the number of articles published. China, which ranks second in the number of articles published (20), lags behind in the average number of citations per article, indicating that it may take more time for China to catch up in terms of citation frequency compared to other countries. Nevertheless, China’s substantial contribution to this field is undeniable (Fig. 3B). The collaborative efforts between countries further underscore the leadership of the United Kingdom and the United States. These collaborations have enhanced the overall quality of research in this field (Fig. 3C, D).

### Contribution of the issuing institution

In bibliometrics, the extent of institutional contribution is often proportionate to the national contribution. However, studying this aspect separately is valuable as it provides insights into the institutions leading research in this field. We visually represented the publishing institutions based on the number of publications (Supplement Figure 1A, Supplemental Digital Content 1, http://links.lww.com/JS9/C207). Among the top five institutions, three are located in the United States, one in China, and one in the United Kingdom, aligning with the top three contributing countries. Notably, although the Second Military Medical University has a higher number of publications (9) compared to Mayo Clinic (7), Mayo Clinic’s publications far outshine other institutions in terms of the average number of citations, averaging at 102 (Supplement Figure 1B, Supplemental Digital Content 1, http://links.lww.com/JS9/C207). This observation underscores the foundation of high-quality research in the United States.

### Source of publications and related field of the research

The source of publication, often referred to as journal contribution, indicates the journals in which these publications are found. The choice of journal frequently reflects the focus of research topics within a specific field. We have compiled a list of 12 journals with more than 3 publications in this field, and the visual results are depicted in Supplement Figure 2A (Supplemental Digital Content 2, http://links.lww.com/JS9/C208). Out of these 12 journals, 6 are related to neurology, while the others cover fields such as trauma and critical care, rehabilitation nursing, spine, and urology. In terms of basic information, the Journal of Trauma-Injury Infection and Critical Care ranks first with five articles. The Journal of Neurology Neurosurgery and Psychiatry boasts the highest impact factor score of 13.654 (Supplement Figure 2B, Supplemental Digital Content 2, http://links.lww.com/JS9/C208), signifying the significance of neurological research. However, research in trauma, critical care, rehabilitation, spine, and urology are also essential for the development of this field. By categorizing and visualizing both the included study publications and their cited documents, we noticed a significant trend in the sources of publications. While the primary sources of the included publications encompassed medicine, neurology, sports, and surgery (Supplement Figure 2C, Supplemental Digital Content 2, http://links.lww.com/JS9/C208), it was noteworthy that molecular biology and genetics emerged as a distinct group. This observation suggests that relying solely on clinical and rehabilitation medicine is insufficient, as basic medical research has played a pivotal role in advancing this field.

### Keywords clustering and future research hotspots

**Figure 4:**
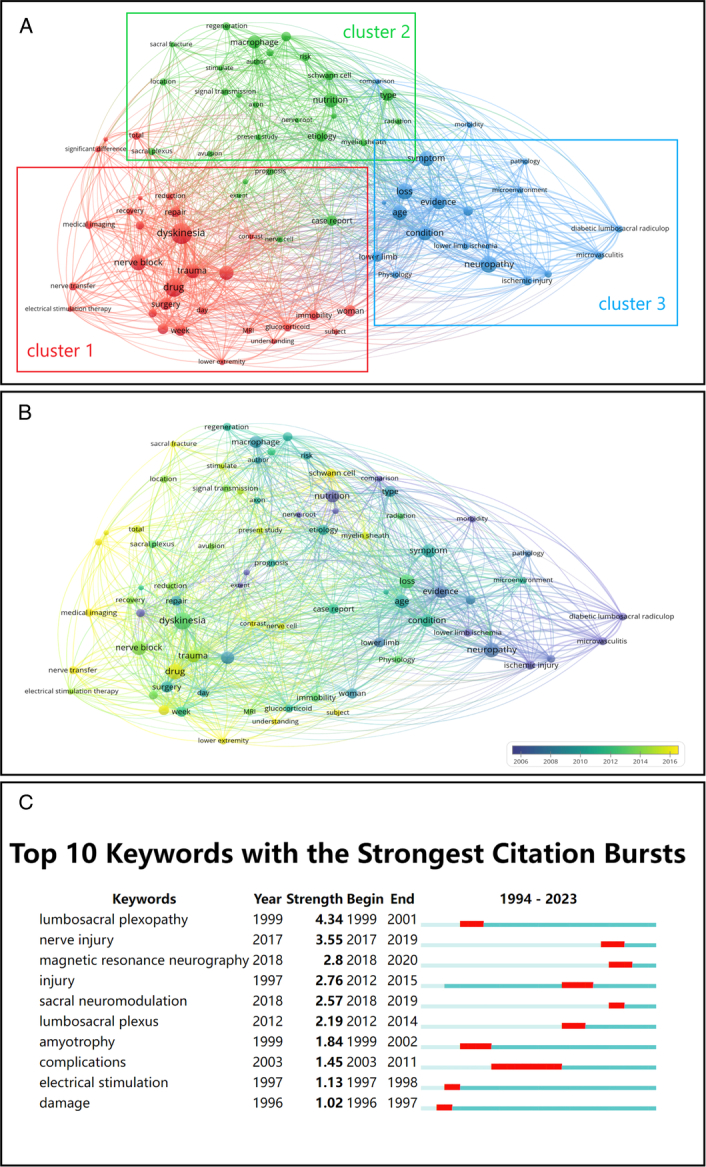
Analysis of keywords in all publications. (A) Mapping of the keywords in the field of lumbosacral plexus injury. The size of the circle represents the frequency of the keyword, and different colors represent different clusters. (B) Distribution of keywords according to the average time of appearance. Blue represents an early appearance, and yellow represents a late appearance. (C) Top 10 keywords cited most frequently, which have received continuous attention for a period of time. The red bars represent frequently cited keywords during this time period.

The analysis of keywords is a pivotal aspect of utilizing bibliometrics to comprehend lumbosacral plexus injury. After conducting a macro-level analysis of key related fields, it becomes essential to identify which keywords will become focal points for future research. We categorized 77 keywords that appeared more than 5 times across 150 publications (Supplement Table 1, Supplemental Digital Content 3, http://links.lww.com/JS9/C209). The results were grouped into three clusters, each representing different facets of the research field. As depicted in Figure 4A: The red cluster (Cluster 1) involves etiology, diagnosis, and treatment, featuring keywords like MRI (7 times), myelography (9 times), glucocorticoid (9 times), nerve transfer (9 times), and electrical stimulation therapy (6 times). The green cluster (Cluster 2) pertains to molecular, cellular, and mechanistic aspects, including keywords such as Schwann cell (10 times), nerve growth factor (6 times), myelin sheath (8 times), axon (7 times), and signal transmission (8 times). The blue cluster (Cluster 3) is associated with physiology and pathology, encompassing keywords like lower limb ischemia (8 times), diabetic lumbosacral radiculoplexus neuropathy (8 times), ischemic injury (10 times), and microvasculitis (9 times). In addition to keyword analysis within these fields, we also assessed the recent attention each field has received. As illustrated in Figure 4B, the lighter areas primarily concentrate in Clusters 1 and 2. This indicates that keywords within Clusters 1 and 2 are relatively new and suggests that research related to etiology, diagnosis, treatment, as well as molecular, cellular, and mechanistic aspects, will form the core of future studies. Among these clusters, the most recent keyword is ‘nerve transfer’ (2015.7778), signifying that nerve transfer will be a focal point of future research. CiteSpace also analyzes keywords, primarily focusing on core keywords that have been prominent over a specific period. In the recent period, we observed that the keyword with the highest heat is ‘sacral neuromodulation’, drawing attention from 2018 to 2019 (Fig. 4C). Research in this particular hotspot is expected to remain a focus in the future.

## Discussion

### Research trends and hotpots about Lumbosacral plexus injury

In summary, the number of publications in the field of lumbosacral plexus injury is expected to continue rising over the next 30 years, indicating a growing interest in this area. When it comes to contributions, the United States, China, and the United Kingdom are the leading countries in terms of the quantity of publications. However, in terms of research quality, the United States stands out significantly, which may be attributed to renowned institutions like Mayo Clinic. Collaboration between countries plays a crucial role in enhancing the quality and quantity of publications. Similarly, interdisciplinary collaboration is vital, with clinical medicine, rehabilitation medicine, molecular biology, and genetics needing to progress together. In clinical medicine, aside from neurology, research in trauma, critical care, rehabilitation, spine, and urology is of utmost importance. Collaborative efforts across various fields can propel the development of this field. Keyword analysis revealed that the future research focus in this field centers on clinical diagnosis and treatment, with keywords like ‘nerve transfer’ and ‘sacral neuromodulation’ taking center stage. These two keywords are central to the clinical management of this condition.

Notably, the symptoms that have the significant impact on the quality of life for lumbosacral plexus injury patients are related to urinary and bowel function. Sacral neuromodulation is a treatment method that involves applying electrical pulses to the sacral nerves to stimulate effector organs like the bladder, urethral sphincter, and anal sphincter, thereby restoring their normal function^10–12^. Numerous clinical studies have demonstrated the significant clinical efficacy of this treatment method. It is characterized by its safety, minimally invasive nature, and effectiveness^13,14^. This indicates a growing focus on managing clinical complications associated with lumbosacral plexus injury, offering promising prospects for patients with defecation disorders.

Nerve transfer emerges as a pivotal approach in the repair of lumbosacral nerve injuries. This method often becomes the last lifeline for functional recovery in patients who have not responded to medical conservative treatment or commonly used surgical techniques like nerve repair or nerve loosening. For a period, the autologous obturator nerve was the preferred choice^15^, yielding favorable outcomes. Many patients experienced a return of lower limb movement to grade 3 or higher without compromising thigh adduction function^15^. Furthermore, the transplantation of healthy L3 combined with S1 nerve root has proven to be effective. This approach was initially tested in rhesus monkeys and subsequently applied in clinical practice, delivering encouraging results for patients^16,17^. Nonetheless, there is still a long road ahead in this field of research. The remodeling of brain function after nerve transplantation will be will the core of research, breakthroughs in this domain could potentially revolutionize neural repair.

### Clinical diagnosis and treatment of lumbosacral plexus injury

Through VOSviewer’s cluster analysis, we conducted a comprehensive examination of the three primary clusters of keywords, our research emphasis shifted towards Clusters 1 and 2, specifically focusing on etiology, diagnosis, and treatment, molecular and cellular mechanisms, where we conducted in-depth exploration and analysis.

**Figure 5:**
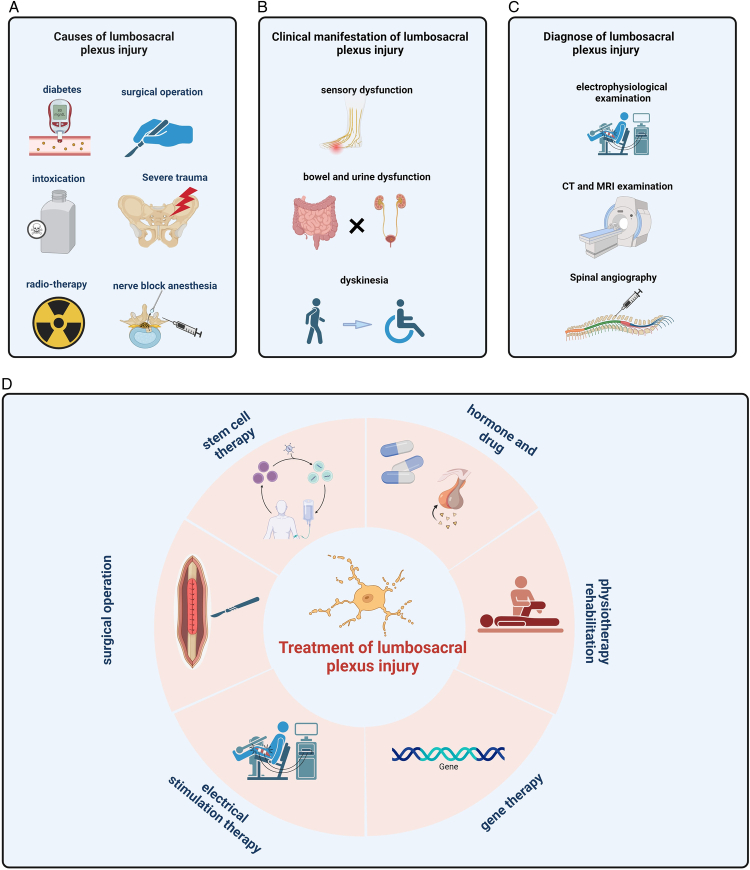
Diagnosis and treatment of lumbosacral plexus injury. (A) Causes of lumbosacral plexus injury. (B) Clinical manifestation of lumbosacral plexus injury. (C) Diagnosis of lumbosacral plexus injury. (D) Treatment of lumbosacral plexus injury.

To begin, concerning the etiology, diagnosis, and treatment of lumbosacral plexus injury, it is essential to consider the factors contributing to the onset of this condition. Lumbosacral plexus injury rarely occurs without identifiable causes, and understanding the core pathogenic factors is crucial. Currently, the prominent factors contributing to this condition include violent trauma, surgical errors, pelvic radiotherapy, lower extremity nerve anesthesia, vascular damage, and diabetic nerve damage (Fig. 5A). In cases of violent trauma, the pelvis is often affected, posing a significant threat to the nerves. Surgical procedures follow a similar principle, as excessive stretching can physically harm the nerves. Pelvic radiotherapy is often a necessary treatment for various pelvic tumors. These tumors have close proximity to the sacral plexus, and the damage caused by radiotherapy can lead to nerve cell necrosis and impairment of nerve function. During neuroanesthesia, the inhibitory effects of anesthesia on synaptic transmission can result in signal loss. Anesthesia that excessively suppresses synapses may lead to global inhibition. Consequently, in cases where the sacral plexus is in a compromised state, a failure to accurately administer the anesthetic dose may result in permanent nerve damage. Additionally, nerve damage is frequently observed in patients with diabetes in clinical practice. These patients often experience limb numbness due to hyperglycemia, which can increase sorbitol production, affecting nerve cells and signal transmission^18–21^.

Patients with lumbosacral plexus injury typically exhibit clinical symptoms, including lower extremity motor and sensory dysfunction, urinary and bowel disorders, and male erectile dysfunction (Fig. 5B). These manifestations arise due to the intricate anatomical structure of the lumbosacral plexus, which encompasses the lumbosacral plexus and sacral plexus, governing all sensory and motor functions of the lower limbs. Additionally, the perineal sensory and motor areas are under the control of the lumbosacral plexus. Consequently, injuries to this region result in a wide range of clinical symptoms.

However, due to the type of injury often accompanied by severe multiple injuries, the rate of missed diagnosis is higher in early diagnosis, the final diagnosis of patients often relies on auxiliary examinations, electrophysiological testing, MRI, and myelography (Fig. 5C). Electrophysiological examination is a classic method where the injured nerve exhibits relatively slow processing and transmission of electrical stimulation, indicative of impaired nerve cell function, which is the gold standard for diagnosing nerve damage. More precise evaluation methods have been introduced in recent years. MRI offers distinct advantages for examining nervous system lesions, boasting high soft tissue resolution and sensitivity to changes in water content within tissue components. This allows for clear visualization of nerve appearance, facilitating rapid diagnosis. In recent years, functional magnetic resonance imaging (FMRI) has made the diagnosis of the damaged plane easier. Similarly, myelography involves the use of contrast media to enhance image clarity^22–26^. In summary, accurately diagnosing lumbosacral plexus injuries necessitates a comprehensive assessment that considers the cause of injury, clinical manifestations, and auxiliary examinations. This approach is pivotal for effective treatment. In the future, it is very important to determine the specific segments of lumbosacral plexus nerve injury (L2-S4) and it is also very important to distinguish the injuries of preganglionic neurons and postganglionic neurons. These diagnostic problems are still unresolved.

Treatment of lumbosacral plexus injuries presents a greater challenge compared to diagnosis. Incomplete lumbosacral plexus injury can be treated by conservative treatment, fracture reduction, decompression, and release. However, the previous treatment effect of lumbosacral plexus complete injury is often pessimistic, and most patients can only give up, and in recent years, this field has developed rapidly. Nerve repair is an exceedingly intricate process, and the field offers a variety of treatment approaches.

For incomplete lumbosacral nerve injury, nonsurgical therapies play important roles, including hormone stimulation, neurotrophic drugs, electrical stimulation, and physical therapies like acupuncture^26–28^. More advanced treatments such as gene and stem cell therapy have also come to the forefront^29^ (Fig. 5D). However, it is important to note that these complementary treatments do not address the underlying problem. Surgical treatment also provides the idea of treatment. Neurolysis is often employed to alleviate physical compression. It frequently involves procedures such as bone reduction, muscle, and fascia clearance to address the issue^30^. Laparoscopic lumbosacral plexus decompression has gradually received more attention in recent years, which provides a new idea for neurolysis^31^. However, this method is not suitable for all lumbosacral plexus segment. These approaches focus on creating a conducive environment for nerve self-repair and accelerating nerve growth through various forms of stimulation. For complete nerve injury and nerve avulsion injury, in recent years, nerve transfer surgery has gained attention. Our bibliometric research has also highlighted the growing interest in nerve transfer. Studies have confirmed that contralateral nerves and the obturator nerve and even the intercostal nerve serve as viable alternatives^32^, The use of healthy L3 combined with the S1 nerve root to repair the injured lumbosacral plexus nerve on the affected side has proven to be effective. This approach not only preserves the function of the healthy side but also promotes functional recovery on the affected side^16^. The effectiveness of brain function remodeling is also very important for the recovery after nerve transplantation. It holds significant promise, making autologous nerve transplantation a compelling treatment option for the future. However, the poor recovery of distal limb function after nerve transplantation, the specific manifestation is the function of the hip and knee joints recovered well, but the function of the ankle joints was poor, which are problems that need to be solved in the future.

Upon delving into the relevant disciplines within this field, the study of molecular, cellular, and mechanistic aspects emerges as an important cluster of keywords. This exploration allowed us to examine the neural repair process from a molecular and cytological perspective, revealing its exceptional complexity. Currently, the recognized neural repair process comprises two primary pathways. The first pathway is collateral sprouting, observed in 10–20% of nerve repair cases, primarily driven by compensatory mechanisms. However, this mechanism is typically not applicable to most clinical cases, particularly those involving amputation injuries.

**Figure 6:**
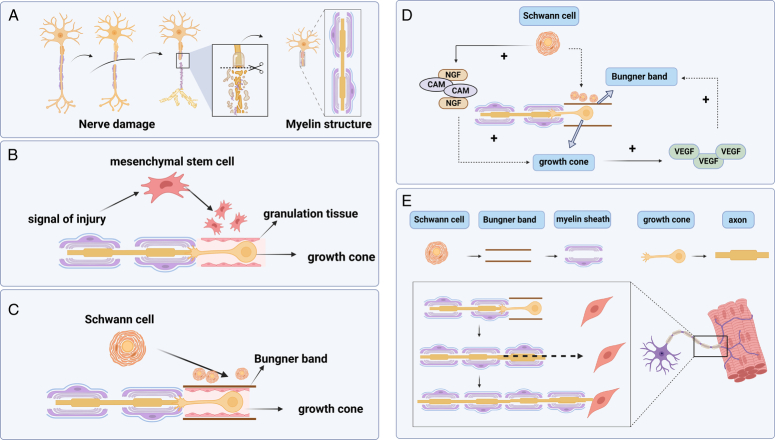
Mechanisms of neural repair. (A) The process of nerve damage. (B–E) Biological processes of nerve repair and reconstruction.

The more prevalent repair mechanism, accounting for 80% of nerve repair cases, is referred to as axon remodeling^33^. When a nerve is severed, the segments below the cut break down, with macrophages responsible for removing cell debris (Fig. 6A). Subsequently, new nerve fibers sprout from the proximal axis and extend toward the distal end, forming what is known as the growth cone. Mesenchymal stem cells proliferate in response to injury signals, creating granulation tissue that guides the growth cone’s extension (Fig. 6B). Additionally, to expedite axon growth cone extension, Schwann cells establish a Bungner band within the granulation tissue, facilitating faster nerve fiber growth from the axis. The axon growth cone releases cell growth factors, while Schwann cells release nerve growth factor (NGF), neurotrophin-3 (NT-3), and cell adhesion molecules that mutually promote growth (Fig. 6C, D)^34,35^. During this process, the nerve elongates, extending its path to reach the neuromuscular junction and perform functions. However, when the nerve injury is too close to the cell body, irreversible neuronal damage may occur, resulting in permanent loss of function. Therefore, at present, the process of neuronal death, the speed of nerve growth and the functional recovery of distal effectors are the contents that need attention in molecular biology research.

Based on the in-depth research and development of neural repair mechanism, more and more new materials for neural repair are being developed. Therefore, new techniques with greater potential are worthy of attention. In recent years, some new biomaterials have been developed to provide insight for peripheral nerve injury. The combination of common hydrogel loading technology, 3D printing technology and nanotechnology may be prospective. For example, electrospun nanofiber nerve conduits have shown great advantages in nerve repair and multifunctional biodegradable conductive hydrogel can improve the neural repair microenvironment by regulating stem cells^36–38^. Even though a large number of *in vivo* and *in vitro* researches have confirmed the effectiveness of some biomaterials, it is still a long process before it can be used in clinical patients.

Although medical treatment and surgical treatment play important roles, they cannot solve the problem of lumbosacral plexus injury completely until now. In the field of rehabilitation medicine, the idea of magnetic stimulation of nerve function is put forward, which can use magnetic field to stimulate sacral nerve to control the contraction of pelvic floor muscle group. It has been shown to be useful in the treatment of urinary incontinence and urinary retention^39^. Whether the combination of electrical stimulation and magnetic stimulation has a better effect still needs more in-depth exploration in the future.

In recent years, translational medicine is the focus of research, and it is an important direction to apply new technology to clinical practice to solve disease problems. At present, one of the most promising technologies is the brain-computer interface (BCI), which can establish a direct communication and control channel between the human brain and the computer, and replace the function of damaged nerves to some extent^40^. As early as 1924, the clinical application of electroencephalography marked the beginning of the BCI, after 100 years of development, existing brain-computer interface technology can help paralyzed patients stand, walk and even climb stairs, which is a boon for patients with lumbosacral plexus damage, especially for those who have failed medical and surgical treatment^41^.Meanwhile, this technology is also one of the closest technologies to clinical patients, and has great clinical application potential.

Therefore, for clinical patients, medical treatment and surgical treatment are the basis of lumbosacral plexus injury, and the progress of biomaterial science, basic medicine and rehabilitation medicine is equally important. Brain-computer interface has great potential and will play an important role in the future.

## Limitations

This research carries potential limitations. Firstly, regarding the database selection, a more comprehensive approach involving multiple databases like Embase and PubMed would have been preferable, but this would require advanced bibliometric techniques. Secondly, the exclusion of non-English literature may have an impact on the overall results. Lastly, the accuracy of bibliometrics in predicting future development trends may be subject to bias due to the inherent nature of bibliometrics. Despite the thorough research and analysis in this paper, we anticipate the emergence of more high-quality articles in this field.

## Conclusions

Over the next 30 years, the research interest in lumbosacral plexus injuries is projected to steadily increase. The United States has made significant contributions to this field, highlighting the importance of international collaboration. Clinical medicine and sports rehabilitation medicine play pivotal roles in this research, with molecular biology and genetics forming the fundamental basis for its development. Within clinical medicine, the study of neurology, trauma, severe illness, rehabilitation, spine, and urology holds utmost significance. In recent years, the research focus has shifted from etiology, prognosis, pathology, and physiology towards clinical diagnosis and treatment. Human nerve transplantation and sacral nerve stimulation are emerging as the latest core keywords and are poised to be central in future research within this field.

## Ethical approval

Not applicable.

## Consent

Not applicable.

## Sources of funding

Not applicable.

## Author contribution

S.W., D.X., D.S.: collected and analyzed the data; S.W.: wrote the manuscript; N.L. and A.C.: designed the study and revised manuscript. All authors revised the manuscript and approved the final manuscript.

## Conflicts of interest disclosure

The authors declare that there is no conflicts of interest.

## Research registration unique identifying number (UIN)

Not applicable.

## Guarantor

Sheng Wang is the guarantor of the entire study.

## Data availability statement

Data can be obtained by supplement information.

## Provenance and peer review

Not applicable.

## Supplementary Material

**Figure:**
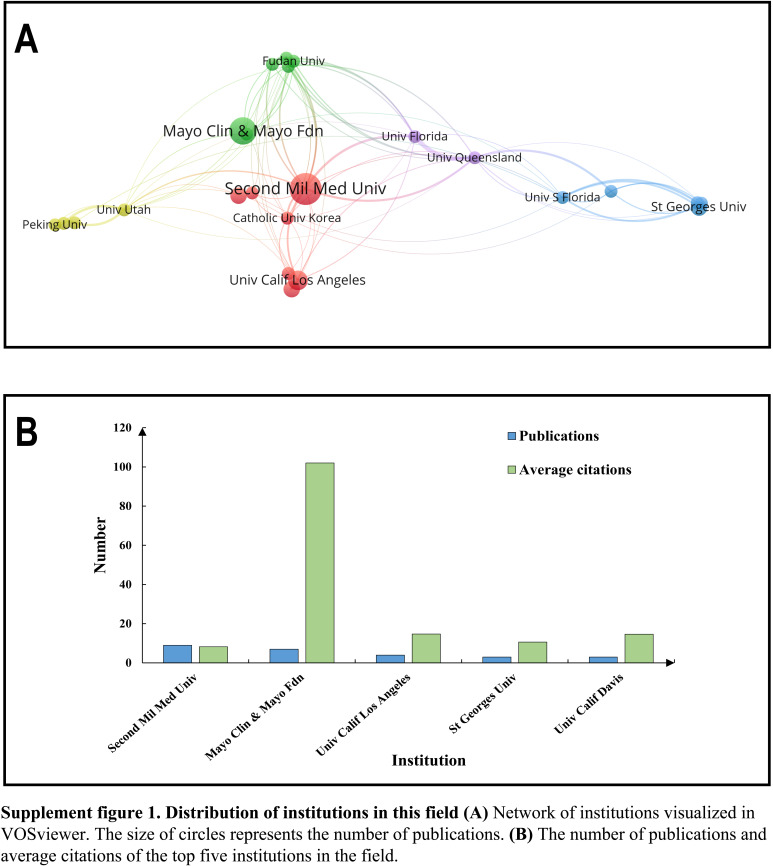


**Figure:**