# Supplementary material for: Could nerve transplantation be the future of this field: a bibliometric analysis about lumbosacral plexus injury
Source: Int J Surg. 2024 Mar 21;110(6):3734–44. doi: 10.1097/JS9.0000000000001332 (PMC11175794; doi:10.1097/JS9.0000000000001332)
Supplement: Supplementary file 1 [file js9-110-3734-s003.docx]

**77 keywords that appear more than 5 times**

| label | weight<Occurrences> | score<Avg. pub. year> |
| --- | --- | --- |
| lumbosacral nerve root avulsion | 6 | 2000.6667 |
| extent | 5 | 2002.6 |
| spinal cord injury | 9 | 2002.6667 |
| ischemic injury | 10 | 2004.3 |
| neuromuscular junction | 6 | 2004.3333 |
| microvasculitis | 9 | 2004.8889 |
| morbidity | 7 | 2005 |
| lower limb ischemia | 8 | 2005.5 |
| diabetic lumbosacral radiculoplexus neuropathy | 8 | 2005.625 |
| nerve root | 6 | 2005.8333 |
| comparison | 6 | 2006 |
| nutrition | 15 | 2006.1333 |
| degeneration | 8 | 2006.625 |
| evidence | 15 | 2007.1333 |
| neuropathy | 18 | 2007.3333 |
| pathology | 7 | 2007.5714 |
| lower limb | 11 | 2007.7273 |
| control | 10 | 2008.1 |
| spinal cord | 15 | 2008.6 |
| woman | 12 | 2008.75 |
| type | 13 | 2008.8462 |
| macrophage | 14 | 2008.9286 |
| author | 7 | 2009 |
| risk | 9 | 2009.3333 |
| repair | 11 | 2009.4545 |
| day | 9 | 2009.5556 |
| surgery | 13 | 2009.9231 |
| age | 16 | 2009.9375 |
| symptom | 16 | 2010.0625 |
| etiology | 12 | 2010.1667 |
| prognosis | 8 | 2010.25 |
| autophagy | 10 | 2010.4 |
| glucocorticoid | 9 | 2010.6667 |
| axon | 7 | 2010.7143 |
| regeneration | 9 | 2010.7778 |
| median | 5 | 2010.8 |
| microenvironment | 7 | 2010.8571 |
| condition | 15 | 2011 |
| case report | 11 | 2011.2727 |
| week | 12 | 2011.6667 |
| neural transplantation | 5 | 2011.8 |
| relationship | 8 | 2012 |
| loss | 17 | 2012.5882 |
| sacral plexus | 8 | 2012.625 |
| radiation | 7 | 2012.7143 |
| dyskinesia | 22 | 2012.7727 |
| immobility | 10 | 2012.8 |
| peripheral nerf | 5 | 2012.8 |
| Physiology | 8 | 2012.875 |
| myelography | 9 | 2013.3333 |
| MRI | 7 | 2013.5714 |
| signal transmission | 8 | 2013.625 |
| avulsion | 7 | 2013.7143 |
| reduction | 8 | 2013.75 |
| recovery | 8 | 2013.75 |
| nerve block | 17 | 2013.7647 |
| location | 8 | 2013.875 |
| electrical stimulation therapy | 6 | 2014.1667 |
| trauma | 15 | 2014.4 |
| nerve growth factor | 6 | 2014.5 |
| stimulate | 6 | 2014.8333 |
| present study | 7 | 2014.8571 |
| total | 9 | 2015.1111 |
| myelin sheath | 8 | 2015.125 |
| nerve cell | 7 | 2015.2857 |
| sacral fracture | 6 | 2015.3333 |
| drug | 19 | 2015.4211 |
| branch | 11 | 2015.4545 |
| contrast | 6 | 2015.5 |
| lower extremity | 7 | 2015.5714 |
| neural restoration | 5 | 2015.6 |
| subject | 6 | 2015.6667 |
| medical imaging | 9 | 2015.7778 |
| nerve transfer | 9 | 2015.7778 |
| significant difference | 7 | 2016 |
| schwann cell | 10 | 2016.8 |
| understanding | 6 | 2019.6667 |
| lumbosacral nerve root avulsion | 6 | 2000.6667 |
